# PointPainting: 3D Object Detection Aided by Semantic Image Information

**DOI:** 10.3390/s23052868

**Published:** 2023-03-06

**Authors:** Zhentong Gao, Qiantong Wang, Zongxu Pan, Zhenyu Zhai, Hui Long

**Affiliations:** 1 Aerospace Information Research Institute, Chinese Academy of Sciences, Beijing 100190, China; 2 Key Laboratory of Technology in Geo-Spatial Information Processing and Application System, Chinese Academy of Sciences, Beijing 100190, China; 3 School of Electronic, Electrical and Communication Engineering, University of Chinese Academy of Sciences, Beijing 101408, China

**Keywords:** deep learning, 3D object detection, multi modal, data fusion, semantic segmentation

## Abstract

A multi-modal 3D object-detection method, based on data from cameras and LiDAR, has become a subject of research interest. PointPainting proposes a method for improving point-cloud-based 3D object detectors using semantic information from RGB images. However, this method still needs to improve on the following two complications: first, there are faulty parts in the image semantic segmentation results, leading to false detections. Second, the commonly used anchor assigner only considers the intersection over union (IoU) between the anchors and ground truth boxes, meaning that some anchors contain few target LiDAR points assigned as positive anchors. In this paper, three improvements are suggested to address these complications. Specifically, a novel weighting strategy is proposed for each anchor in the classification loss. This enables the detector to pay more attention to anchors containing inaccurate semantic information. Then, SegIoU, which incorporates semantic information, instead of IoU, is proposed for the anchor assignment. SegIoU measures the similarity of the semantic information between each anchor and ground truth box, avoiding the defective anchor assignments mentioned above. In addition, a dual-attention module is introduced to enhance the voxelized point cloud. The experiments demonstrate that the proposed modules obtained significant improvements in various methods, consisting of single-stage PointPillars, two-stage SECOND-IoU, anchor-base SECOND, and an anchor-free CenterPoint on the KITTI dataset.

## 1. Introduction

In 3D object-detection scenarios, vehicles are generally equipped with LiDAR and cameras to acquire point cloud and RGB images. However, the task of handling complex scenarios is arduous using a single sensor. LiDAR-only methods find it arduous to detect objects that are far from the sensor, since the reflection points are too sparse. In contrast, image-only methods are vulnerable to occlusion and bad weather, such as fog and snow. Therefore, multi-modal approaches that use both sensors have become a popular research direction. Recently, many new multi-modal methods [1,2,3,4] have been proposed. However, LiDAR-only methods, such as SE-SSD [5] and PV-RCNN [6], outperform these in the KITTI [7] 3D object-detection benchmark. This anomaly indicates the importance of finding an effective fusion strategy to improve the 3D object-detector performance.

PointPainting [8] proposes a fusion strategy that attaches the semantic scores of the RGB image to the LiDAR points based on the transformation relationship between the image and the point cloud. This can be applied to various existing LiDAR-only methods and requires minimal changes to the network architecture. However, in some cases, the detection accuracy of PointPainting [8] drops compared with the original methods. Therefore, this paper proposes PointPainting++, including the following methods, to solve the problems in PointPainting [8].

This paper first proposes a novel anchor weight-assignment strategy to reduce the negative impact of inaccurate semantic information. Inaccurate semantic information is generated due to the following three points. Firstly, the semantic result of the image is imperfect due to the quality of the segmentation algorithm. In addition, there are errors in the calibration of cameras and LiDARs. Finally, rounding operations are needed during the transformation between the pixels and LiDAR points, which also introduces errors.

**Figure 1:**
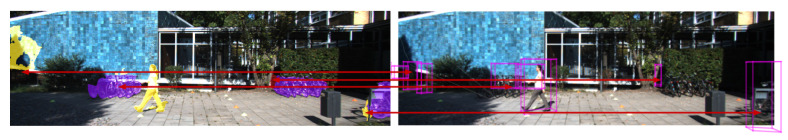
Semantic segmentation and 3D object-detection results of painted PointPillars [8] on the KITTI [7] dataset. The purple and yellow parts in the semantic segmentation results represent motorcycle and pedestrian categories, respectively. The inaccurate parts of the semantic segmentation results lead to false detections.

As shown in Figure 1, these inaccurate segmentations often correspond to many false detections, contrary to the intention of introducing semantic information. In this paper, we propose a strategy that uses the proportion of inaccurate points contained in the anchor to generate their weights in the loss function. In this way, anchors with more inaccurate points will play a more critical role, enhancing the detector’s ability to distinguish ambiguous objects.

Furthermore, a dual-attention module based on the SEBlock [9] is introduced to the detection network. This module measures the importance of the channels and points in a voxelized point cloud and generates weights for each voxel in the channel and point dimensions. This module can suppress channels with inaccurate semantic information for each LiDAR point on the one hand, and suppress the features of LiDAR points carrying inaccurate semantic information on the other.

**Figure 2:**
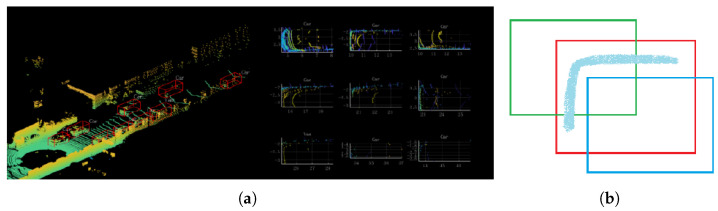
Figure (**a**) is a annotation sample from the KITTI [7] dataset, and Figure (**b**) is a typical case of anchor assignment. The red box in Figure (**b**) is the ground-truth box, and the green and blue boxes are anchors. A positive anchor tag will be assigned to the blue box by the max-IoU assigner. However, the blue box containing few target LiDAR points is not a high-quality positive anchor.

In addition, a SegIoU-based anchor assigner is used for more efficient anchor assignment. As shown in Figure 2a, the distribution of LiDAR points is often concentrated on the surface, since they are obtained by collecting the reflected laser. This phenomenon is more pronounced for objects of larger sizes, such as cars and trucks. Figure 2b shows that both boxes are assigned positive anchor tags when using the max-IoU assigner [10]. However, the blue box does not contain any target points, which makes classification difficult. This shows that the max-IoU assigner will introduce many controversial positive anchors with few points inside when processing “L”-shaped LiDAR points. We proposed a SegIoU-based anchor assigner to only assign positive tags to anchors that have a high degree of overlap and similar semantic information with ground-truth boxes. In this way, the inferior positive anchors containing few target points will be screened out due to different semantic information with ground-truth boxes.

These three improvements are evaluated on the KITTI [7] 3D and bird’s-eye view (BEV) object-detection benchmarks. The results showed that PointPainting++ could better improve the performance of cars, pedestrians, and cyclists compared with PointPainting [8]. Experiments on the KITTI [7] *valid* set proved that our strategy is effective in multiple methods.

**Contributions.** PointPainting++ combines image and LiDAR point information more effectively based on PointPainting [8] and reduces detector interference caused by inaccurate semantic information. Our contributions are as follows:

- **Anchor weight-assignment strategy.** We propose a way to assign weights to anchors based on semantic information. The detector becomes more discriminative by paying more attention to the problematic anchors carrying more inaccurate semantic information.
- **Dual-attention module.** We adopt a dual-attention module to enhance the voxelized point cloud. This module suppresses the inaccurate semantic information in a voxelized point cloud.
- **SegIoU-based anchor assigner.** We use a SegIoU based anchor assigner to filter out abnormal positive anchors, to avoid confusion and improve detector performance.

## 2. Related Works

### 2.1. Multi-Modal 3D Object-Detection Methods

**Figure 3:**
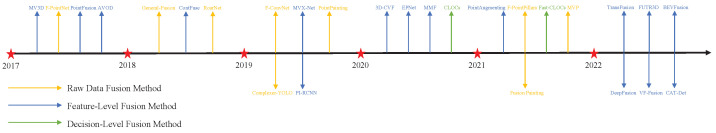
Chronological overview of the multi-modal 3D object-detection methods.

According to the level of data fusion, current multi-modal 3D object-detection methods using both point cloud and RGB images can be divided into three categories: raw data fusion, feature-level fusion, and decision-level fusion. A chronological overview of the multi-modal 3D object-detection approaches is shown in Figure 3.

#### 2.1.1. Raw Data Fusion

The raw data fusion-based method fuses RGB images and LiDAR points before they are fed into a detection pipeline. Such methods are generally built sequentially: 2D detection or segmentation networks are first employed to extract information from the RGB images, and then the extracted information is passed to the point cloud, and finally the enhanced point cloud is fed to the point-cloud-based detectors. Based on the fusion types, the raw data fusion-based methods can be divided into two categories: region-level fusion and point-level fusion.

**Region-level fusion.** Region-level fusion methods aim to utilize information from RGB images to constrain object candidate regions in the point cloud data. Specifically, an image is first passed through a 2D object detector to generate a 2D bounding box. Then, the bounding boxes are extruded into 3D viewing frustums. Finally, the LiDAR points within the frustums are sent to the point-cloud-based detector. F-PointNet [11] first proposes this fusion mechanism, and many new methods have been proposed to improve this fusion framework. Representative methods of this category include F-ConvNet [12], RoarNet [13], F-PointPillars [14], and General-Fusion [15].

**Point-level fusion.** Point-level fusion methods aim to enhance point cloud data with image information. The enhanced point cloud is then fed into a point-cloud-based detector for better detection results. PointPainting [8] is the pioneer of such methods. This fusion strategy has been followed by a lot of papers, including Fusion-Painting [16], Complexer-YOLO [17], and MVP [18].

#### 2.1.2. Feature-Level Fusion

The feature-level fusion-based method builds fused features using the features extracted from the point cloud and images. This method is currently the most popular multi-modal method and many fusion methods fall into this category, since traditional CNN is not available on raw point clouds. The feature fusion methods can be divided into three categories based on the fusion stages [19].

**Fusion in backbone.** Such methods first correspond the LiDAR points to the pixels through a transformation between the camera coordinate system and the LiDAR coordinate system. After that, the features from a LiDAR backbone and the features from an image backbone using various fusion operators are fused according to this pixel-to-point correspondence. This fusion strategy can be performed in the middle layers of a voxel-based detection backbone. Representative methods included MMF [20], MVX-Net [21], DeepFusion [22], and CAT-Det [23]. In addition, this fusion strategy can also be conducted only at the feature maps of the voxel-based detection backbone. Representative methods include 3D-CVF [1], FUTR3D [24], BEVFusion [25], VF-Fusion [26], TransFusion [27], and PointAugmenting [28]. In addition to the fusion in voxel-based backbones, there also exist some papers incorporating image information into the point-based detection backbone, including PointFusion [29], EPNet [3], and PI-RCNN [2].

**Fusion in proposal generation and RoI head.** In such methods, 3D object proposals are first generated from a LiDAR detector, and then the 3D proposals are projected onto the image view and bird’s-eye view to crop features from the image and LiDAR backbone, respectively. Finally, the cropped image and LiDAR features are fused in an RoI head to predict parameters for each 3D object. MV3D [30] and AVOD [31] are pioneers using multi-view aggregation for multi-modal detection. FUTR3D [24] and TransFusion [27] employ the transformer [32] decoder as the RoI head for multi-modal feature fusion.

#### 2.1.3. Decision-Level Fusion

Decision-level fusion merges the results of a LiDAR-based network and an image-based network at the decision level. It does not need to consider the interaction of the point cloud and RGB image at the information level, resulting in low complexity. The representative methods include CLOCs [4] and Fast-CLOCs [33].

### 2.2. PointPainting

**Figure 4:**
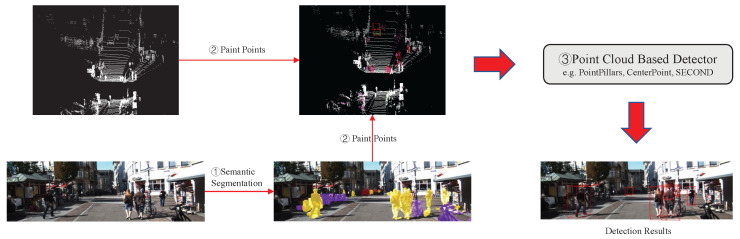
Architecture of PointPainting [8]. This consists of three main stages: (1) image-based semantic segmentation, (2) point cloud painting, and (3) point-cloud-based detector.

PointPainting [8] is one of the raw data-fusion methods and is the basis of the method proposed in this paper. As shown in Figure 4, the architecture of PointPainting [8] consists of three main stages: (1) semantic segmentation: an image-based semantic segmentation network that generates pixel-wise semantic scores; (2) point cloud painting: painting LiDAR points with the semantic scores; (3) point-cloud-based detector: a point-cloud-based 3D object-detection network with changed input channels. The three stages will be described in detail in the following sections.

#### 2.2.1. Semantic Segmentation

The image-based semantic segmentation network takes an RGB image as input and outputs a matrix containing the predicted class scores that correspond to all pixels. These scores contain rich semantic information which can complement the point cloud. PointPainting [8] can use the existing semantic segmentation module to complete this step.

#### 2.2.2. Point Cloud Painting

The data-fusion method of PointPainting [8] is shown in Algorithm 1. A LiDAR point can be projected onto an RGB image by an affine transformation. PointPainting [8] finds the corresponding pixel of the LiDAR point on the RGB image based on this transformation and then attaches the semantic scores of the pixel to the LiDAR point, forming new channels.
**Algorithm 1** Point Cloud Painting(L,S,T).
**Inputs:**   LiDAR point cloud $L\in R^{N,D}$ with N points, *D* features. Segmentation scores $S\in R^{W,H,C}$ with *C* categories. Homogeneous transformation matrix $T\in R^{4,4}$.**Output:**   Painted LiDAR points $L_{p}\in R^{N,D+C}.$1:**for** l∈L **do**
2:   $\left(x_{img},y_{img}\right)=\mathrm{PROJECT}\left(T,l\right)$3:   $s=S[x_{img},y_{img},:]$
4:   p=Concatenate(l,s)5:**end for**$⊳l_{\mathrm{img}}\in R^{1,2}$$⊳s\in R^{1,C}$$⊳p\in R^{1,D+C}$

Take the KITTI [7] dataset as an example. The calibration file of the KITTI dataset gives the intrinsic matrix $P_{i}\in R^{4,4}$ of camera *i*, the correction matrix of camera 0 $R_{\mathrm{rect}}^{0}\in R^{4,4}$, and the projection matrix between the LiDAR and camera coordinate system ${\mathrm{Tr}}_{\mathrm{velo}}^{\mathrm{cam}}\in R^{4,4}$. A LiDAR point $n\in R^{4,1}$ (homogeneous coordinates) can be projected onto the camera *i* image using the following formula:

(1) $$m=P_{i}\times R_{\mathrm{rect}}^{0}\times {\mathrm{Tr}}_{\mathrm{velo}}^{\mathrm{cam}}\times n$$

where $m\in R^{4,1}$ (homogeneous coordinates) represents the coordinates of the projected point in the camera coordinate system. The transformation in the above formula can be represented by a homogeneous transformation matrix $T\in R^{4,4}$. Thus, the above formula can also be expressed as:

(2) m=T×n

Each LiDAR point in the KITTI [7] dataset is (x,y,z,r), where (x,y,z) is the spatial location of each point and *r* is the reflectance of each point. The output of the semantic segmentation network is *C* class scores $\left(s_{0},s_{1},s_{2},\cdots ,s_{C-1}\right)$, where C=4 (car, pedestrian, cyclist, background). Once the LiDAR points are projected to the image, the semantic scores of the relevant pixel $\left(x_{img},y_{img}\right)$ are appended to the LiDAR point (x,y,z,r) to generate the painted LiDAR point $\left(x,y,z,r,s_{0},s_{1},s_{2},s_{3}\right)$.

#### 2.2.3. Point-Cloud-Based Detector

The point-cloud-based detectors of different structures can be adapted to detect objects with painted points, simply by changing their input dimension. Better detection results can be achieved due to this additional semantic information.

## 3. PointPainting++

In this section, the details of PointPainting++ are introduced, followed by the efficient acceleration algorithm that this process uses.

### 3.1. PointPainting++ Architecture

**Figure 5:**
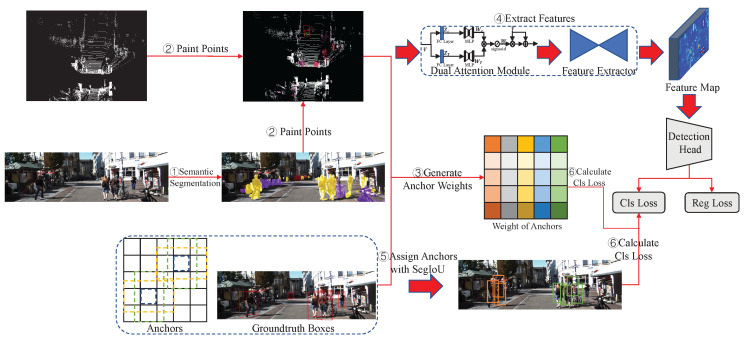
Architecture of PointPainting++. It consists of six steps: (1) image-based semantic segmentation, (2) point cloud painting, (3) generation of anchor weights, (4) feature extraction, (5) SegIoU-based anchor assignment, (6) calculation of classification loss.

As shown in Figure 5, the main architecture of PointPainting++ consists of six steps. In the first and second steps, we follow PointPainting [8] to attach semantic information to the LiDAR points. In the third step, the weight of each anchor is generated by counting the proportion of inaccurate points and the total points inside each anchor. Then, the voxelized point cloud is weighted using the dual-attention module, followed by feature extraction using the backbone of the point-cloud-based method. After that, a SegIoU-based assigner is used to assign anchors. Finally, the classification loss is calculated by the anchor assignment result and the weight of each anchor.

The following sections will detail our improvements to PointPainting [8].

#### 3.1.1. Anchor Weight Assignment

In this paper, we propose a strategy for assigning weights to each anchor during the calculation of classification loss. Points containing inaccurate semantic information need to be labeled before weights are assigned. As shown in Algorithm 2, a LiDAR point will be considered to be an outlier if its semantic information does not match the ground truth. Different labels will be appended to the end of LiDAR points to distinguish them.

**Figure 6:**
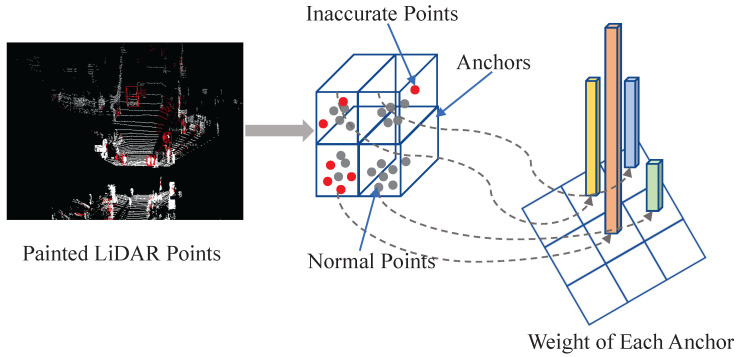
Anchor weight-assignment strategy. The weight of each anchor is calculated according to the proportion of inaccurate points it contains.

After labeling the inaccurate points, as shown in Figure 6, each anchor will be assigned a weight according to the proportion of inaccurate points in it. The more inaccurate points are contained within an anchor, the harder classification becomes. Therefore, the more inaccurate points an anchor contains, the higher weight it is assigned. The specific formula is as follows:

(3) $$w=\alpha +\beta \times \frac{N_{inaccurate}}{N_{total}+\xi }$$

**Algorithm 2** Mark Points(L,G).
**Inputs:**   Painted LiDAR point $L\in R^{N,D+C}$ with N points, *D* features, *C* categories. Ground-truth boxes $G\in R^{M,F}$ with *M* boxes, *F* encoding features; the last dimension represents the category.**Output:**   Augmented LiDAR points $L_{p}\in R^{N,D+C+1}.$1:**for** l∈L **do**2:   s=L[D+1:]3:   $p_{s}=\mathrm{Argmax}\left(s\right)$4:   **for**  gt∈G  **do**5:     **if** l in gt and $p_{s}\neq \mathrm{gt}\left[-1\right]$ **then**6:        p=Concatenate(l,1)7:     **end if**8:   **end for**9:   **if** $p_{s}\neq 0$ **then**10:     p=Concatenate(l,1)11:   **else**12:     p=Concatenate(l,0)13:   **end if**14:**end for**   $⊳s\in R^{1,C}$   $⊳p\in R^{1,D+C+1}$   $⊳p\in R^{1,D+C+1}$ $⊳p\in R^{1,D+C+1}$  

In the above formula, α is the base weight of each anchor, β is the additional weight coefficient, ξ is a small number preventing the denominator from being zero, and $N_{inaccurate}$, $N_{total}$ represent the number of inaccurate points and the total number of points in the anchor, respectively. The weight assigned to each anchor ranges from α to α+β and linearly increases with the proportion of inaccurate points within the anchor. In this way, the difficult anchors that contain more inaccurate semantic information will play a more important role in the classification loss. The detector will also pay more attention to the anchors that are difficult to classify and obtain the ability to distinguish controversial samples, thereby showing better performance.

Such a weighting strategy is suitable for both anchor-based and center-based methods. Take the PointPillars [34] and CenterPoint [35] as examples.

The loss function of PointPillars [34] consists of the classification loss $L_{cls}$, the location regression loss $L_{loc}$, and the direction loss $L_{dir}$:

(4) $$L=\frac{1}{N_{pos}}\left(\beta_{loc}\times L_{loc}+\beta_{cls}\times L_{cls}+\beta_{dir}\times L_{dir}\right)$$

where $N_{pos}$ is the number of positive anchors and $\beta_{loc},\beta_{cls},\beta_{dir}$ are the weight coefficients of these three losses, respectively. The classification loss can be weighted according to the strategy in this section:

(5) $$L_{cls}=\sum_{i}w_{i}\times L_{cls}^{i}$$

where $w_{i}$ is the weight of each anchor, generated as mentioned above.

Similarly, the loss function of CenterPoint [35] consists of the heatmap loss $L_{hm}$ and the location regression loss $L_{reg}$:

(6) $$L=\frac{1}{N_{pos}}\left(\beta_{hm}\times L_{hm}+\beta_{reg}\times L_{reg}\right)$$

where $N_{pos}$ is the number of positive anchors and $\beta_{hm},\beta_{reg}$ are the weight coefficients of these two losses. The detection head of CenterPoint [35] outputs a heatmap, which indicates the probability that there is a target center at this location. Each point on the heatmap corresponds to an area in the original space, and this area can be regarded as a pseudo-anchor when applying the weight-assignment strategy. Thus, the weights of this region can be calculated in the same the way as the anchor weights. The weighted $L_{hm}$ can be expressed as follows:

(7) $$L_{hm}=\sum_{i}w_{i}\times L_{hm}^{i}$$

where $w_{i}$ is the weight of each anchor, generated as mentioned above.

#### 3.1.2. Dual-Attention Module

**Figure 7:**
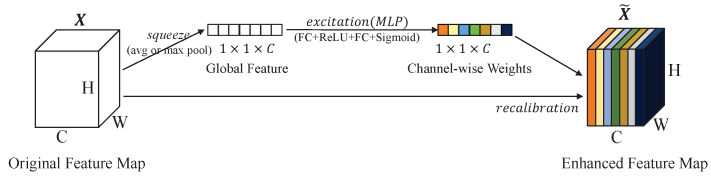
The structure of SEBlock [9]. It first uses the *squeeze* operation to generate global features, and then uses the *excitation* operation to capture channel dependencies and generate channel-wise weights.

In addition to constraining inaccurate features from the perspective of the loss function, we further considered improving the network structure to suppress the inaccurate features. A structure based on SEBlock [9] is proposed to weigh the voxelized point cloud. This combines the channel dimension and point dimension to generate the weights of the voxelized point cloud. The structure of the SEBlock [9] is depicted in Figure 7. For any feature map $X\in R^{H,W,C}$, an SEBlock can be constructed to perform feature recalibration. The features X are first put through a *squeeze* operation, which produces a channel descriptor by aggregating feature maps across their spatial dimensions (H×W). The aggregation is followed by an *excitation* operation, which takes a simple self-gating mechanism that takes the embedding as input and produces a collection of channel-wise weights. These weights are applied to the feature map X to generate the output of the SEBlock [9], which can be directly fed into subsequent layers of the network.

**Figure 8:**
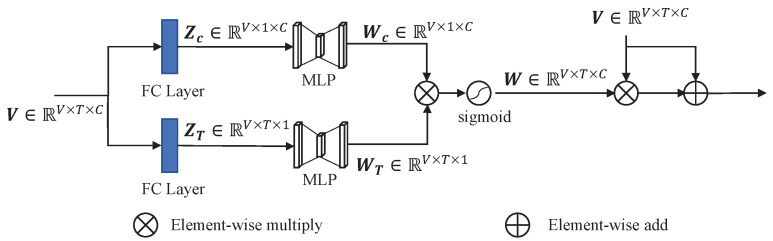
The architecture of the dual attention module. This module has a symmetrical structure, and each part can be regarded as an SEBlock [9]. Fully connected layers are used to compress dimensions to extract global features.

As shown in Figure 8, this module consists of two SEBlocks [9]. For any voxelized point cloud $V\in R^{V,T,C}$ (*V* voxels, *T* points in each voxel, *C* features of each point), we use the fully connected layer to compress the channel dimension and point dimension, respectively, to extract global features. The global features then undergo a simple gating mechanism to generate weights for channel and point dimensions:

(8) $$\begin{array}{cc} W_{c} & =F_{ex}\left(Z_{c},W\right)=W_{2}\left(\delta \left(W_{1}\left(Z_{c}\right)\right)\right)\\ W_{T} & =F_{ex}\left(Z_{T},W^{\prime }\right)=W_{2}^{\prime }\left(\delta \left(W_{1}^{\prime }\left(Z_{T}\right)\right)\right) \end{array}$$

δ refers to the ReLU function, $W_{1}\in R^{\frac{C}{r},C},W_{2}\in R^{C,\frac{C}{r}},W_{1}^{\prime }\in R^{\frac{T}{r},T}$ and $W_{2}^{\prime }\in R^{T,\frac{T}{r}}$. We parameterize the gating mechanism to limit model complexity by forming a bottleneck with two fully connected (FC) layers. Subsequently, the element-wise multiply operation is used to comprehensively consider both channel and point dimensions and obtain the final weight through a sigmoid activation:

(9) $$W=\sigma (W_{c}\cdot W_{T})$$

Finally, the original features V and weighted features W·V are combined through the element-wise add operation and fed into the subsequent network. The weight W comprehensively considers the weight of each point in a voxel and the weight of each channel in a point. On the one hand, the weight of each point suppresses the features of points with inaccurate semantic information in the voxel. On the other hand, the weight of each channel suppresses the features of channel with wrong semantic information in each point. Therefore, the inaccurate semantic information is suppressed after the dual attention module, and the subsequent part of the detector can obtain more accurate features, thus showing performance improvement.

#### 3.1.3. SegIoU-Based Anchor Assigner

As mentioned in Section 1, many anchors that contain few target points are assigned positive tags. In order to remove those controversial positive anchors, we propose the SegIoU-based anchor assigner.

We follow the anchor-assignment strategy of faster R-CNN [10] to assign a binary class tag (of being an object or not) to each anchor. Two kinds of anchors are assigned a positive tag: (1) the anchor/anchors with the highest IoU with a ground-truth box, (2) an anchor with an IoU that is higher than the positive threshold with any ground-truth box. Anchors with an IoU that is lower than the negative threshold to any ground-truth box are assigned a negative tag. Anchors that are neither positive nor negative do not contribute to classification loss.

On this basis, SegIoU is proposed instead of IoU for anchor assignment, which considers the degree of overlap between anchors and ground-truth boxes both in geometry and semantics:

(10) $$SegIoU\left(P,Q\right)=IoU\left(P,Q\right)-\gamma \times H\left(S_{p},S_{q}\right)$$

where γ is a hyperparameter used to control the numerical size of the H(p,q), $S_{p}$ and $S_{q}$ represent the semantic scores of points inside anchor and ground-truth box, respectively, and $H(S_{p},S_{q})$ is the Hellinger distance used to quantify the similarity between the two probability distributions. $S_{p}$ and $S_{q}$ are obtained by averaging the semantic scores of the points within the anchor and ground-truth box. Most anchors do not contain any points inside due to the spareness of the point cloud. The semantic scores of such anchors are set to a uniform distribution.

After obtaining the probability distribution, the Hellinger distance $H(S_{p},S_{q})$ can be computed as:

(11) $$H(S_{p},S_{q})=\frac{1}{\sqrt{2}}\sqrt{\sum_{i=0}^{n-1}{\left(\sqrt{s_{i}^{p}}-\sqrt{s_{i}^{q}}\right)}^{2}}$$

The semantic information of the ground-truth boxes usually has certain categories. Therefore, the probability distribution of the ground truth boxes will differ from those of the controversial positive anchors, making the semantic loss of controversial anchors more prominent. Thus, the SegIoU of the controversial anchors will be lower than that of the normal positive anchors. The controversial anchors can be filtered from positive anchors with a threshold that is set in advance.

The SegIoU-based anchor assigner has strict rules, which often result in a low number of positive anchors. We adopted an insurance mechanism to avoid the over-screening problem. Let $n_{seg}$ be the number of positive anchors selected by the SegIoU-based assigner and $n_{iou}$ be the number of positive anchors selected by the max-IoU assigner. We set the minimum value of the number of positive anchors $n_{min}$ to $0.7\times n_{iou}$. The anchors will be sorted by SegIoU if $n_{seg}<n_{min}$, and the top $n_{min}$ ones will be selected as positive anchors.

### 3.2. An Efficient Acceleration Algorithm

Without exception, the methods mentioned in the previous section need to analyze the points in each anchor. For example, the number of inaccurate points and total points inside each anchor are needed when assigning weights to anchors. However, the anchors are generated according to the feature map, and each location on the feature map corresponds to anchors of different sizes, which means that the number of anchors is usually large. The number of anchors can be calculated by the following formula:

(12) N=W×H×C

where *W* and *H* represent the size of the feature map, and *C* represents the number of anchor categories corresponding to each point on the feature map. Given a 400×400 feature map, if there are three types of targets to be detected and each type has two orientations, then the total number of anchors will be 400×400×3×2 = 960,000. Using the traversal and loop to handle such a massive number of anchors will consume a large amount of computational resources and severely slow down the training speed.

We propose utilizing 2D convolution to speed up this process. The size of anchors in the z-direction can be ignored because the anchor settings in the z-direction include all the parts in which points exist. A feature map with the same size as the voxelized point cloud can be constructed to record the information needed in each voxel after this simplification. Finally, after a 2D convolution of this feature map using the convolution kernel corresponding to the anchor, a tensor that records the information needed in each anchor can be obtained.

**Figure 9:**
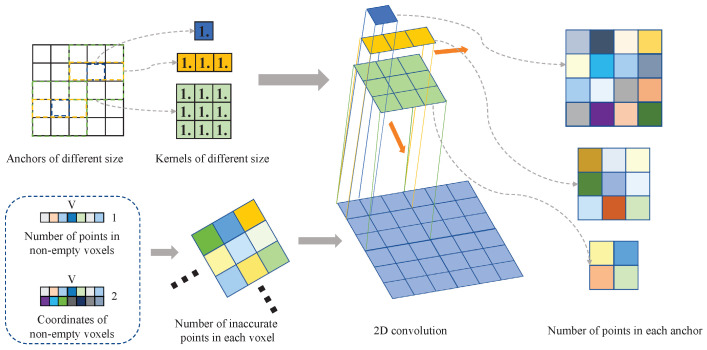
The 2D convolution calculation process for the number of points in each anchor. The size of the convolution kernels is determined by the size of anchors. We generated a tensor that records the number of points in each voxel first, and then used different convolution kernels to perform 2D convolution on this tensor to obtain the number of points in each anchor.

A case in point is the calculation of the number of points in each anchor. We illustrate this calculation process in Figure 9. This process can be divided into three steps. The first step is to voxelize the LiDAR points and obtain three tensors $V\in R^{V,T,C},N\in R^{V,1}$, $C\in R^{V,2}$, which represent the features after voxelization, the number of points in non-empty voxels, and the 2D coordinates of non-empty voxels. *V* represents the number of non-empty voxels, *T* represents the number of points collected within each voxel, and *C* represents the number of features of each point. The tensor that records the number of points inside each voxel can be obtained by filling N according to C into a tensor of the same size as the voxelized point cloud. The second step aims to determine the size of the convolution kernel by calculating the quotient of the anchor size and the voxel size. Finally, the convolution stride is determined by the scaling factor of the point cloud feature. The number of points in each anchor can be calculated by performing 2D convolution using the convolution kernel filled with 1.0 on the tensor that records the number of points in each voxel. Such convolution operations can be quickly accelerated by GPU, significantly improving the training speed compared with loop operations.

## 4. Experimental Setup

In this section, we present the details of the dataset and the experimental setup for PointPainting++.

### 4.1. Dataset and Evaluation Metrics

We evaluated PointPainting++ on the KITTI [7] dataset. The data acquisition platform of the KITTI [7] dataset contained 2 grayscale cameras, 2 color cameras, 1 LiDAR, 4 optical lenses, and 1 GPS navigation system. Synchronized point cloud and images from the left and right color cameras in the KITTI [7] dataset were adopted. The dataset contained 7481 training samples and 7518 testing samples, with a total of 80,256 labeled objects. Three types of objects were detected, as required by the KITTI [7] object-detection benchmark: *car, pedestrian, cyclist.* We followed the guidance of [30,31] to further divide the training data into two groups, 3712 data and 3769 validation data, according to the partition file for experimental evaluation. We also followed the standard practice [30,36] to not use points projected outside the image range for training, since the ground-truth boxes are only annotated within the image range.

The results were evaluated using average precision (AP) as an indicator containing the IoU thresholds for three classes. The evaluation was conducted at three levels of difficulty—easy, moderate, and hard—according to the occlusion level, maximal truncation, and the height of the 2D box in the corresponding image.

### 4.2. Semantic Segmentation Network

We used DeepLabV3+ [37], implemented by mmsegmentation https://github.com/open-mmlab/mmsegmentation (accessed on 20 May 2021), for semantic segmentation. The module was trained on the CityScapes [38] dataset, which is similar to the KITTI [7] image data scene. We kept the semantic scores of cars and pedestrians, and followed PointPainting [8] to generate cyclists’ semantic scores. The semantic scores of the background category were obtained by adding the semantic scores of the other categories.

The image data collected by camera 2 and camera 3 were used for semantic segmentation. Any LiDAR point was discarded if its projection fell outside the perception range of camera 2 or 3. The semantic information of each LiDAR point contained the average value of the semantic scores of the images collected by the two cameras.

### 4.3. Point-Cloud-Based Network

We used the public code OpenPCDet https://github.com/open-mmlab/OpenPCDet (accessed on 17 January 2023) for PointPillars [34], SECOND [39], CenterPoint [35] and SECOND-IoU [39]. These existing methods cover the most common network structures: one-stage and two-stage, anchor-base and center-base, and voxel-feature and pillar-feature. Experiments show that PointPainting++ has good generality and improves the performance of networks of various architectures. Based on the original code, we implemented a new dataset—painted KITTI, instead of KITTI [7]—for experiments. The point cloud in painted KITTI contains expanded information, including the semantic scores of the four categories and accurate flag information. This changes the dimensions of point cloud from 4 to 9. The expanded point cloud is easily accepted by many existing LiDAR backbones after the input dimensions are changed. To compare this with PointPainting [8], the accurate flag information was not used in the training process. The SegIoU-based anchor assigner is only valid for anchor-based methods, including PointPillars [34], SECOND [39], and SECOND-IoU [39].

## 5. Experimental Results

This section describes the experimental results of PointPainting++ on the KITTI [7] dataset.

### 5.1. Quantitative Analysis

PointPainting++ was evaluated on various detection networks with different structures, including PointPillars [34], SECOND [39], CenterPoint [35] and SECOND-IoU [39]. We compared PointPainting++ with the original network and PointPainting methods in both 3D and BEV object-detection tasks. For the easy, moderate, and difficult samples, the IoU thresholds of the car category were 0.7, 0.5, and 0.5, respectively, and the IoU thresholds of the other categories were all 0.5. The mean average precision (mAP) over three kinds of different difficulty levels was used to represent the overall performance of the method. The fusion versions of each network that use PointPainting [8] will be referred to as being painted (e.g., Painted PointPillars), while the fusion versions that use PointPainting++ will be referred to as being painted++ (e.g., Painted PointPillars++).

As shown in Table 1, PointPainting++ showed a significant performance improvement for both 3D and BEV mAP compared to PointPainting [8] on detection networks with different structures. PointPainting [8] showed a performance degradation on some networks (e.g., SECOND-IoU [39]) due to the interference of inaccurate semantic information. Table 1 illustrates that the SECOND-IoU [39] after using PointPainting++ not only shows a performance improvement on the basis of PointPainting [8], but also achieves a better performance than the original network.

As shown in Table 2, Table 3, Table 4 and Table 5, PointPainting++ showed a significant performance improvement in the detailed detection results for each category. As mentioned in PointPainting [8], for narrow vertical objects such as pedestrians, which are indistinguishable when using only LiDAR points, the introduction of semantic information leads to a more significant performance improvement. A further analysis of the experimental results is as follows:

Compared to PointPainting [8], after larger weights were assigned to anchors containing inaccurate semantic information, the network showed a significant performance improvement in the pedestrian category. In addition, PointPainting++ can also improve the performance degradation of certain categories mentioned in PointPainting [8]. Table 2, Table 3, Table 4 and Table 5 shows that PointPainting++ achieved a better performance than PointPainting [8] on all categories, and can achieve better results than the point-cloud-based network on certain structured detectors. This shows that our method can make more effective use of semantic information compared to PointPainting [8].

### 5.2. Ablation Study

We also incrementally added three improvements to the network. In the following discussion, we refer to the improved methods adopted by PointPainting++ as: I. anchor weight assignment with semantic information; II. dual-attention module; III. anchor-assignment strategy based on semantic information. PointPillars [34] was adopted as the benchmark method for this experiment. As shown in Table 6, the performance of the PointPillars [34] improved in the car and cyclist categories, a drop in performance was shown in the pedestrian category after I was applied. This is because the semantic results for pedestrians are usually more accurate, while those of cyclists contain errors. The detector will pay more attention to the cyclist category, which contains more inaccurate information under the guidance of weights. In addition, the performance of the PointPillars [34] in the pedestrian category was significantly improved after the addition of the dual-attention module, while also maintaining the improvements in the car and cyclist categories that were achieved in the previous step. Furthermore, the performance of the PointPillars [34] improved in all three categories after the SegIoU-based anchor assigner was introduced. The improved performance of the network demonstrates the effectiveness of PointPainting++.

### 5.3. Qualitative Analysis

**Figure 10:**
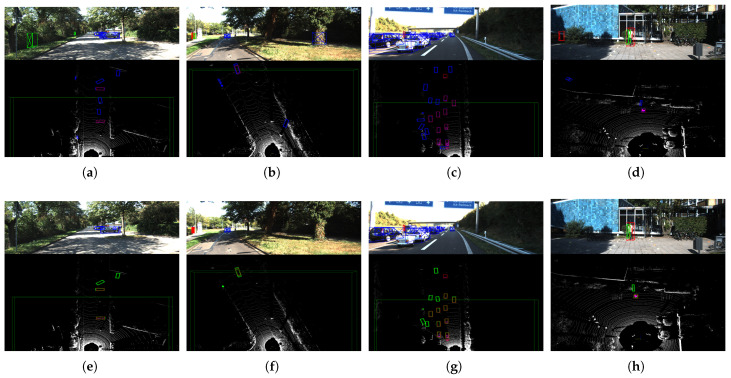
Qualitative results of our PointPainting++ of PointPillars [34], CenterPoint [35], SECOND [39], and SECOND-IoU [39] on the KITTI [7] *valid* set. The upper part of each picture is the 3D detection projected to the image, and the lower part is the 3D detection in the LiDAR point cloud. The blue, green, and red boxes in the 2D detection results represent the car, cyclist, and pedestrian categories, respectively. The red boxes in the 3D result are the ground-truth boxes, and the rest of the boxes are the detection boxes. The results indicate that PointPainting++ improves the performance of detectors of various structures in multiple scenarios. (**a**) Painted PointPillars [34], (**b**) Painted CenterPoint [35], (**c**) Painted SECOND [39], (**d**) Painted SECOND-IoU [39], (**e**) Painted PointPillars++, (**f**) Painted CenterPoint++, (**g**) Painted SECOND++, (**h**) Painted SECOND-IoU++.

Figure 10 shows the qualitative results of our PointPainting++, as applied to Painted PointPillars [34], CenterPoint [35], SECOND [39] and SECOND-IoU [39]. In Figure 10a, the original Painted PointPillars [8] wrongly detects cyclists in the bushes on the side of the road, while Figure 10e shows that our PointPainting++ eliminates these false detection results. In addition, as shown in Figure 10b, false detections remain, although PointPainting [8] helps in the detection of vertical narrow objects on the ground. However, as shown in Figure 10f, our PointPainting++ eliminated two such false detections. Figure 10c shows a scene with many overlapping targets. There are often numerous false detections in such scenarios, since many non-target points are projected to the target pixel positions. Figure 10g indicates that our PointPainting++ effectively reduces these false detections. Finally, as shown in Figure 10d, false detections may occur due to the inaccurate semantic results contained in single-target scenarios. In contrast, Figure 10h shows the performance improvement in our PointPainting++ in this scenario.

**Figure 11:**
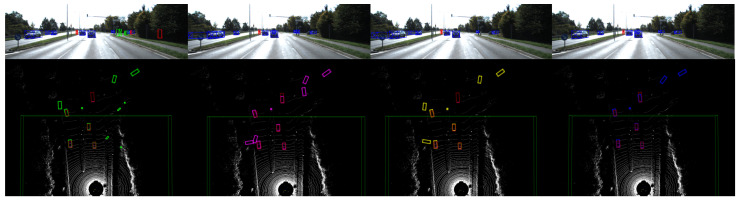
Qualitative results of our three improvements in the Painted PointPillars++. The experimental results of the Painted PointPillars [8] obtained with different improvement methods are shown from left to right. The blue, green, and red boxes in the 2D detection results represent the car, cyclist, and pedestrian categories, respectively. The red boxes in the 3D result are the ground-truth boxes, and the rest of the boxes are the detection boxes. False detections were significantly reduced as the improvements were introduced.

Figure 11 shows the qualitative results of our PointPainting++ when applied to Painted PointPillars [34]. The false detections in the multi-objective scene continued to decrease after improvement measures were applied to Painted PointPillars [34], which shows that the three improvement measures adopted in our PointPainting++ have a positive effect, reducing false detections and improving network performance.

In sum, the qualitative analysis results show that PointPainting++ improves performance compared with existing methods in various network structures and various scenarios. The improvement methods all have a positive effect on reducing false detections and improving the performance of the object detector.

## 6. Discussions

Here, we performed ablation studies on the KITTI [7] *valid* dataset. All studies used the Painted PointPillars architecture and all parameters were kept constant except for the research objects.

### 6.1. The Influence of Anchor Weight

PointPainting++ reduces the confusion caused by inaccurate semantic information by assigning larger weights to anchors with more inaccurate LiDAR points. The effectiveness of this method strongly depends on the correct weight settings. The main reasons for this are as follows: most of the loss functions in the existing methods adopt the form of focal loss [40], which can pay more attention to difficult anchors. The network may pay too much attention to difficult anchors if the weights of difficult anchors are too large. In addition, we do not offer special treatment for empty anchors, which also have a strong confounding effect. Assigning too much weight to non-empty anchors may make it complex for the network to classify such anchors correctly.

To explore the impact of the anchor weights on network performance, we conducted ablation experiments with the following settings: α in Equation (3) was set to the constant 1.0. Thus, the relative size of the weights of anchors with inaccurate semantic information can be adjusted by changing β.

**Figure 12:**
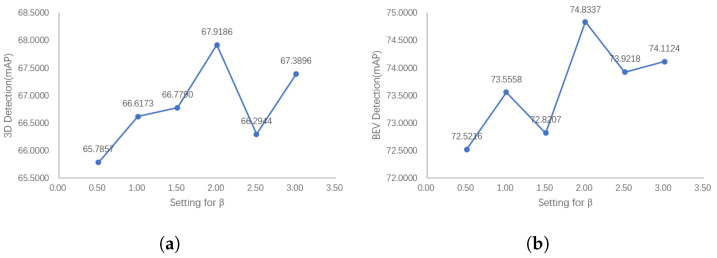
The 3D and BEV detection results of our PointPainting++ with the relative weight coefficient β. The detection performance first reaches the peak with the increase in β, and then shows a downward trend. If weights are too large, the detector will focus too much on the difficult samples, while weights that are too small will not emphasize the difficult samples. (**a**) 3D detection results with β (**b**) BEV Detection results with β.

Figure 12 shows the results of our ablation experiments. Figure 12 indicates that the detection results of 3D and BEV show a similar trend; that is, with the increase in β, the detector performance reaches a peak value, and then decreases with the increase in β. This is because when β is small, assigning larger weights to difficult anchors with inaccurate semantic information can cause the network to pay more attention to difficult anchors, but when β is too large, this is counterproductive for the reasons mentioned above.

### 6.2. The Influence of Semantic Weight in SegIoU

Our PointPainting++ further measures the degree of overlap between anchors and ground-truth boxes by adding semantic loss to the IoU and sifting out inferior positive anchors that contain only a few target LiDAR points. Therefore, it is particularly important to control the relative size of the loss of semantic information. On the one hand, it will be unable to filter out the inferior positive anchors if the semantic loss term is too small. On the other hand, if the semantic loss term is too large, there will be too few positive anchors, reducing the performance of the network. We tuned the relative size of the semantic loss term in SegIoU by changing the hyperparameter γ in Equation (10).

**Figure 13:**
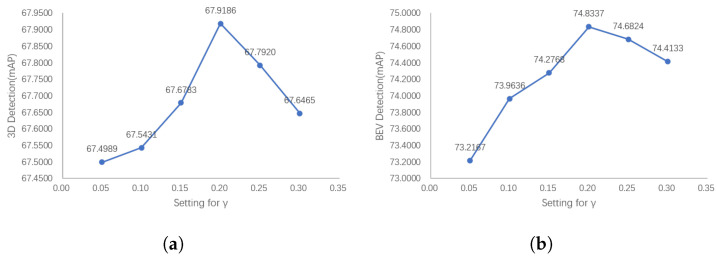
The 3D and BEV detection results of our PointPainting++ with the semantic loss coefficient γ. The detector performance shows a trend of increasing and then decreasing as the semantic loss items increase. If the semantic loss terms are too large, there will be too few positive anchors, while semantic loss terms that are too small will not filter inferior positive anchors using semantic information. (**a**) 3D Detection results with γ (**b**) BEV detection results with γ.

Figure 13 shows that the performance of the detector first shows an upward trend with the increase in the semantic loss weight in SegIoU, and then shows a downward trend after reaching the peak. This is consistent with the previously mentioned reasons, and also shows that the choice of an appropriate semantic loss size in the actual training process plays an important role in improving the detector performance.

### 6.3. The Influence of the Number of Positive Anchors

It is difficult to intuitively quantify the impact of the semantic loss term in the process of using SegIoU to filter out positive anchors, which often results in over-screening and a decrease in the detector performance. A moderate threshold is needed to limit the minimum number of positive anchors. On the one hand, the over-screening problem cannot be solved if the threshold is too small. On the other hand, the desired purpose of screening out inferior positive anchors will not be achieved if the threshold is too large. We changed this threshold and conducted ablation experiments to explore the effect of this threshold setting on the detector performance.

**Figure 14:**
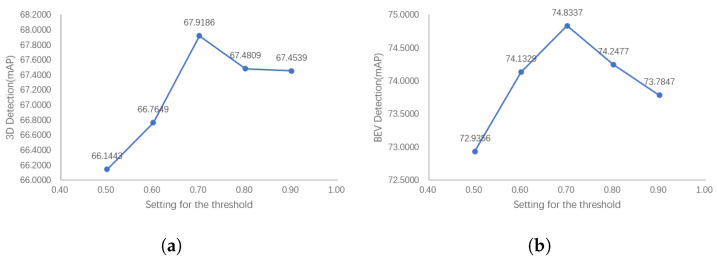
The 3D and BEV detection results of our PointPainting++ with the number of positive anchors. The detection performance peaks after an appropriate number of positive anchors is selected. The excessive retention of the max-IoU assigner-based results may fail to remove inferior positive anchors, while having too few positive anchors will make it difficult for the detector to learn target features. (**a**) 3D detection results with the number of positive anchors. (**b**) BEV detection with the number of positive anchors.

Figure 14 shows the results of our ablation experiments. The detection results show that the performance of the detector first shows an upward trend with the increase in threshold, and starts to decline after reaching the peak. This is consistent with our previous analysis, indicating that the selection of too large or small a threshold will affect the effectiveness of SegIoU and lead to performance degradation.

## 7. Conclusions

In this paper, we propose a new 3D object-detection method based on PointPainting [8]. Three improvements were proposed to address the shortcomings of PointPainting [8]. Firstly, we proposed a weighting strategy for the loss function according to the accuracy of the semantic information, aiming to solve the problem of the point cloud containing inaccurate semantic information. Secondly, a dual-attention module was used to weigh the voxelized point cloud in the channel and point dimensions. Thirdly, we proposed a SegIoU-based anchor-assigner to filter these anchors, which effectively removes inferior positive anchors containing few target points. The experimental results show that our PointPainting++ shows significant performance improvements compared with PointPainting [8] in different network structures and various scenarios. Compared with PointPainting [8], our PointPainting++ does not introduce additional computation in the inference phase and adds very few parameters in the training phase, which means that the training time of the existing network is smaller.In summary, our PointPainting++ can improve the problems in PointPainting [8], and has a certain practical value.

## Figures and Tables

**Table 1:** Comparison of experimental results on the KITTI [7] valid set.

	PointPillars [34]	Painted PointPillars [8]	Painted PointPillars++	Delta
$mAP_{3D}$	66.60	66.30	67.92	+1.62
$mAP_{BEV}$	71.63	73.23	74.83	+1.60
	SECOND [39]	Painted SECOND [8]	Painted SECOND++	Delta
$mAP_{3D}$	67.53	68.24	68.88	+0.64
$mAP_{BEV}$	71.99	73.62	74.01	+0.39
	CenterPoint [35]	Painted CenterPoint [8]	Painted CenterPoint++	Delta
$mAP_{3D}$	67.87	68.25	69.08	+0.83
$mAP_{BEV}$	72.53	72.96	73.96	+1.00
	SECOND-IoU [39]	Painted SECOND-IoU [8]	Painted SECOND-IoU++	Delta
$mAP_{3D}$	70.88	70.78	71.37	+0.59
$mAP_{BEV}$	75.10	75.64	76.26	+0.62

**Table 2:** Comparison of detailed experimental results for each category on the KITTI [7] valid set.

Method	Category	${\mathrm{AP}}_{3D}$			${\mathrm{mAP}}_{3D}$	Category	${\mathrm{AP}}_{\mathrm{BEV}}$			${\mathrm{mAP}}_{\mathrm{BEV}}$
		Easy	Moderate	Hard			Easy	Moderate	Hard	
	Car	87.19	77.46	75.81	**80.15**	Car	89.84	87.46	85.32	**87.54**
PointPillars [34]	Pedestrian	55.13	50.09	46.71	**50.65**	Pedestrian	59.56	55.29	51.74	**55.53**
	Cyclist	82.66	63.91	60.47	**69.01**	Cyclist	84.84	67.68	62.90	**71.81**
	Car	86.83	77.30	75.87	**80.00**	Car	89.61	87.73	86.05	**87.80**
Painted PointPillars [8]	Pedestrian	59.64	55.29	50.81	**55.25**	Pedestrian	66.75	60.77	57.31	**61.61**
	Cyclist	78.52	57.87	54.57	**63.65**	Cyclist	82.59	66.25	62.06	**70.30**
	Car	87.24	77.43	75.65	**80.10**	Car	89.69	87.62	85.77	**87.70**
Painted PointPillars++	Pedestrian	64.87	57.41	52.73	**58.33**	Pedestrian	70.56	63.53	60.04	**64.71**
	Cyclist	80.49	59.29	56.18	**65.32**	Cyclist	84.75	67.47	64.06	**72.09**

**Table 3:** Comparison of detailed experimental results for each category on the KITTI [7] valid set.

Method	Category	${\mathrm{AP}}_{3D}$			${\mathrm{mAP}}_{3D}$	Category	${\mathrm{AP}}_{\mathrm{BEV}}$			${\mathrm{mAP}}_{\mathrm{BEV}}$
		Easy	Moderate	Hard			Easy	Moderate	Hard	
	Car	88.27	78.27	77.05	**81.20**	Car	89.77	87.62	86.21	**87.87**
SECOND [39]	Pedestrian	56.22	52.36	47.04	**51.87**	Pedestrian	59.74	55.02	51.19	**55.32**
	Cyclist	80.27	66.43	61.89	**69.53**	Cyclist	83.54	69.30	65.50	**78.78**
	Car	87.87	77.91	76.63	**80.80**	Car	89.66	87.57	86.36	**87.86**
Painted SECOND [8]	Pedestrian	58.68	53.88	50.67	**54.41**	Pedestrian	62.45	56.12	54.11	**57.56**
	Cyclist	81.21	65.47	61.87	**69.49**	Cyclist	87.59	71.12	61.57	**75.43**
	Car	88.29	78.46	77.23	**81.33**	Car	89.64	87.92	86.74	**88.10**
Painted SECOND++	Pedestrian	58.89	54.06	50.62	**54.52**	Pedestrian	62.57	56.47	54.73	**57.92**
	Cyclist	83.42	66.62	62.30	**70.78**	Cyclist	90.05	70.69	67.28	**76.01**

**Table 4:** Comparison of detailed experimental results for each category on the KITTI [7] valid set.

Method	Category	${\mathrm{AP}}_{3D}$			${\mathrm{mAP}}_{3D}$	Category	${\mathrm{AP}}_{\mathrm{BEV}}$			${\mathrm{mAP}}_{\mathrm{BEV}}$
		Easy	Moderate	Hard			Easy	Moderate	Hard	
	Car	87.16	79.16	76.95	**81.09**	Car	89.03	87.22	85.91	**87.39**
CenterPoint [35]	Pedestrian	55.75	52.84	50.48	**53.02**	Pedestrian	60.00	58.50	55.35	**57.95**
	Cyclist	80.63	66.13	61.71	**69.49**	Cyclist	82.71	69.46	64.59	**72.25**
	Car	87.38	79.48	77.19	**81.35**	Car	89.17	87.57	86.56	**87.76**
Painted CenterPoint [8]	Pedestrian	57.66	54.30	50.71	**54.22**	Pedestrian	61.59	58.60	55.83	**58.67**
	Cyclist	81.99	64.80	60.73	**69.17**	Cyclist	85.86	67.66	63.81	**72.44**
	Car	87.58	79.75	77.34	**81.56**	Car	89.18	87.40	86.88	**87.82**
Painted CenterPoint++	Pedestrian	59.98	55.92	52.81	**56.24**	Pedestrian	63.82	60.84	57.60	**60.75**
	Cyclist	81.07	65.19	62.06	**69.44**	Cyclist	87.65	68.02	64.23	**73.30**

**Table 5:** Comparison of detailed experimental results for each category on the KITTI [7] valid set.

Method	Category	${\mathrm{AP}}_{3D}$			${\mathrm{mAP}}_{3D}$	Category	${\mathrm{AP}}_{\mathrm{BEV}}$			${\mathrm{mAP}}_{\mathrm{BEV}}$
		Easy	Moderate	Hard			Easy	Moderate	Hard	
	Car	89.10	79.11	78.17	**82.13**	Car	90.14	88.12	86.83	**88.36**
SECOND-IoU [39]	Pedestrian	61.45	55.31	50.26	**55.67**	Pedestrian	64.84	58.26	53.98	**59.03**
	Cyclist	86.02	71.56	66.90	**74.83**	Cyclist	89.14	73.61	70.95	**77.90**
	Car	88.63	78.90	77.88	**81.80**	Car	90.13	87.91	86.91	**88.32**
Painted SECOND-IoU [8]	Pedestrian	62.08	55.17	49.78	**55.68**	Pedestrian	66.03	58.33	55.32	**59.89**
	Cyclist	85.81	71.19	67.53	**74.85**	Cyclist	93.74	72.75	69.67	**78.72**
	Car	88.82	78.90	77.85	**81.86**	Car	90.19	88.13	86.98	**88.43**
Painted SECOND-IoU++	Pedestrian	63.83	56.43	50.64	**56.97**	Pedestrian	67.91	59.96	56.18	**61.35**
	Cyclist	86.70	71.58	67.54	**75.27**	Cyclist	93.03	73.46	70.53	**79.01**

**Table 6:** Comparison of experimental results on different, improved methods of PointPainting++ on the KITTI [7] valid set.

Method	Category	${\mathrm{AP}}_{3D}$			${\mathrm{mAP}}_{3D}$	Category	${\mathrm{AP}}_{\mathrm{BEV}}$			${\mathrm{mAP}}_{\mathrm{BEV}}$
		Easy	Moderate	Hard			Easy	Moderate	Hard	
	Car	86.83	77.30	75.87	**80.00**	Car	89.61	87.73	86.05	**87.80**
Painted PointPillars [8]	Pedestrian	59.64	55.29	50.81	**55.25**	Pedestrian	66.75	60.77	57.31	**61.61**
	Cyclist	78.52	57.87	54.57	**63.65**	Cyclist	82.59	66.25	62.06	**70.30**
	Car	86.91	77.35	76.05	**80.10**	Car	89.63	87.72	86.08	**87.81**
Painted PointPillars+I	Pedestrian	56.83	53.96	49.02	**53.27**	Pedestrian	65.24	60.20	57.07	**60.84**
	Cyclist	79.19	57.98	54.26	**63.81**	Cyclist	84.28	67.46	64.07	**71.93**
	Car	86.97	77.35	75.85	**80.06**	Car	89.58	87.62	85.99	**87.73**
Painted PointPillars+II	Pedestrian	60.73	55.86	51.60	**56.06**	Pedestrian	67.67	62.30	58.37	**62.78**
	Cyclist	78.72	59.03	55.22	**64.33**	Cyclist	85.16	67.28	63.71	**72.05**
	Car	87.24	77.43	75.65	**80.10**	Car	89.69	87.62	85.77	**87.70**
Painted PointPillars+III	Pedestrian	64.87	57.41	52.73	**58.33**	Pedestrian	70.56	63.53	60.04	**64.71**
	Cyclist	80.49	59.29	56.18	**65.32**	Cyclist	84.75	67.47	64.06	**72.09**

## Data Availability

Restrictions apply to the availability of these data. KITTI is a project of Karlsruhe Institute of Technology and Toyota Technological Institute at Chicago. It is available at https://www.cvlibs.net/datasets/kitti/ (accessed on 25 February 2023) with the permission.
